# Prevalence and associated factors of depressive symptoms among patients with cancer receiving radiotherapy in southern Thailand: a university hospital-based cross-sectional study

**DOI:** 10.1186/s12904-023-01145-0

**Published:** 2023-03-16

**Authors:** Jarurin Pitanupong, Wannapa Phirom, Rungarun Kittichet, Kanthee Anantapong

**Affiliations:** 1 Department of Psychiatry, Faculty of Medicine, Prince of Songkla University, Hat Yai, Songkhla 90110 Thailand; 2 Division of Radiation Therapy and Oncology, Department of Radiology, Faculty of Medicine, Prince of Songkla University, Songkhla, Thailand

**Keywords:** Associated factor, Cancer, Depression, Pain, Patient, Prevalence

## Abstract

**Background:**

Depression in patients with cancer is frequently underestimated and causes major suffering to patients and families. This study purposed to determine the prevalence of, and factors associated with, depressive symptoms among Thai patients with cancer receiving radiotherapy. The results could promote and optimize the quality of life for patients with cancer.

**Methods:**

A cross-sectional study explored outpatients with cancer at Songklanagarind Hospital; from May to July 2022. The questionnaires inquired about: (1) demographic and personal data, (2) The Patient Health Questionnaire-9 (PHQ-9) Thai version, (3) Verbal Numerical Rating Scale (VNRS), and (4) The meaning in life questionnaire (MLQ). Patient demographics and depressive symptoms were analyzed using the descriptive statistic method and reported as: proportion, frequency, median and interquartile range (IQR). The analysis of the association between independent variables and depressive symptoms was conducted using multiple logistic regression, and performed by R Foundation for Statistical Computing version 4.1.2. All confidence intervals (CIs) were calculated at the 2-sided, 95% level.

**Results:**

There were 113 Thai outpatients with cancer who participated in this study. The majority of them were female (61.1%), and the overall mean age was 56.2 ± 13.6 years. The participants’ cancer staging was stage 2 and 3 (31.0%, and 32.7%, respectively). No participants had experienced major depression (PHQ-9 score of nine or greater), and all of them were mild (23.0%) or no/minimal depression (77.0%). Fifty-four participants (47.8%) were free of pain, with half of them (50.4%) having insomnia. Most of them had a high score in all subparts of meaning in life. The factors associated with mild depression were the history of hospitalization, the perception of one’s health, the presence of physical symptoms, and pain.

**Conclusion:**

In this study, all cancer participants who received radiotherapy had either no/minimal or mild depression. No participants had major depression. Most participants had meaning in their life; however, over half of them still experienced pain and insomnia. To optimize the quality of life, and prevent depression, physical symptoms, and pain should ensure they receive adequate management. Additionally, feeling meaningful in life, and satisfaction in one’s health should also be promoted.

## Background

In patients with cancer, the prevalence of major depressive disorder (MDD) ranges from 15.0 to 29.0%, which is about three to five times greater than in the general population [1, 2]. In the central regions of Thailand, the prevalence of MDD in patients with cancer receiving radiotherapy, assessed by different measurements, ranges from 3.5 to 15.7% [3]. Based on the Diagnostic and Statistical Manual of Mental Disorders (DSM) 5-TR criteria, patients with MDD present with depressive symptoms for at least two weeks. Depressive symptoms consist of: low mood, diminished pleasure in activities, feelings of worthlessness, recurrent thoughts of death, inappropriate or excessive guilt, indecisiveness or diminished ability to think, insomnia, weight loss, decrease in appetite, psychomotor retardation, and fatigue [4]. However, many physical symptoms or complications; such as fatigue, pain [5], sleep disorders [6], impaired physical activity, poor social interaction, reduced decision-making capacity [7, 8], and poor compliance with treatment [9] may result from untreated MDD among patients with cancer. On the other hand, MDD symptoms; such as sleep disruption, fatigue, loss of appetite, and weight loss closely mirror the side effects of cancer treatments; therefore, MDD can be underestimated in clinical practice [10], and this needs clinical attention.

In addition, untreated pain and MDD among patients with cancer are important due to their negative consequences [11] and association with increased complications, resulting in more and longer periods of hospitalizations, and increased healthcare expenses [12]. Although pain and MDD are treatable conditions [13, 14], clinicians have reported a lack of time to perform the diagnostic work as a significant barrier to diagnosing MDD [15]. Recommendations and systemic support that encourage all health cancer care professionals to promptly address the problem of MDD [16, 17] and pain in cancer will be valuable.

Moreover, our prior study identified that 92.7% of Thai patients with palliative cancer preferred to have a sense of being meaningful in life at the end-of-life period [18]. Songklanagarind Hospital, which is the research site of the current study, regularly applies the “narrative” model for the patient with cancer care. The narrative model is used to change the meaning of the illness or cancer by allowing valuable things to happen in their life and to search for meaning in life. As a result, patients’ views of cancer can be shifted from a negative, malignant disease to a more positive one [19]. Thus, the narrative model can be of great value for use in determining the meaning of life and changing the meaning of negative life among patients with cancer and may prevent or reduce depressive symptoms in this population.

The prevalence and associated factors with MDD among patients with cancer may vary across countries and settings; however, existing data are still limited concerning these issues in Thailand. A recent study has been conducted in the central region of Thailand [3]. While the majority of Thailand’s population is Buddhist, most provinces in the southern region are multicultural and some are prominently Muslim. Thus, Southern peoples’ values and beliefs may vary from those of other Thai regions. Hence, this study purposed to determine the prevalence and factors related to MDD among Thai patients with cancer. Moreover, the relationship between meaning in life, the satisfaction of one’s health, and MDD were of interest. These findings might provide valuable knowledge for the establishment of a comprehensive care program aimed at the early detection, prevention, and management of MDD among patients with cancer, as well as promoting the meaning of life of the patients.

## Methods

This cross-sectional study was conducted at Songklanagarind Hospital, Thailand, which is an 800-bed university hospital serving as a tertiary referral center in Southern Thailand. The hospital, together with Songklanagarind Foundation, and Khok Nao Temple, a religious organization, construct the “Yensira” building to accommodate low-economic patients or patients who come from distant provinces and lack relative care, especially patients with cancer who need daily hospital treatments, for example, throughout radiotherapy and chemotherapy.

## Participant selection

All outpatients with cancer treated at the Radiotherapy Clinic; from May to July 2022, were invited to collaborate in this study. To be included, one aged over 20 years of age, had to meet the criteria of being an outpatient with cancer by the owner clinician and being retrieved from the medical register, acknowledge his/her cancer diagnosis, be able to understand and use the Thai language well, agreeing to collaborate in the study and finishing all of the questionnaires. Those who were unknown of their cancer diagnosis and lacked the mental ability to finish all of the questionnaires were excluded.

The command ‘n.for.survey’ in the Epicalc package in R program was used for the sample size calculation (given delta = half of p and alpha = 0.05). In one meta-analysis, the prevalence of MDD among patients with cancer receiving radiotherapy was found to be 12.1%, by the sum-score methods of PHQ-9 [3]. Therefore, the required sample size for our study was 113.

## Participant recruitment

All of the outpatients with cancer who visited the Radiotherapy Clinic were initially screened by the nurse, as to whether they met the inclusion criteria, they were then invited to collaborate in the study. The researchers approached all of them for recruitment and handed them a data sheet, which delineated the rationale for the survey and the allotted time to finish the study. They had at least 15–20 min to deliberate whether to participate in the survey or not. If they wished to collaborate, all of them were requested to sign the informed consent form and were invited to a closet room to finish the questionnaires. During the interview and while performing the interview following the questionnaires, the researcher monitored the participant’s reactions and declared to the participants if they felt uneasy, distressed, or had no willingness to collaborate further then the interview could be stopped at any time. Moreover, if the participants showed a high level of worry or distress, opinion or further planning was offered to them. Additionally, when any participant had a PHQ-9 total score of nine or greater (moderate to severe depression), an appointment to the psychiatric outpatient clinic would be made for them, with the permission of the participants. If the participants had a risk for suicide, they would be sent to consult with a psychiatrist immediately. Additionally, the collected data was used to analyze and for underlying diseases were searched from the Songklanagarind hospital computer program.

## Measures

1. General demographic information inquires around areas associated with age, gender, religion, marital status, education, income, number of household members, type or location of cancer, staging (four stages (I-IV), based on the Tumor-Node-Metastasis (TNM) system), and duration of cancer illness, history of hospital admission, physical and psychiatric illness, perception of physical one’s health, presence of a physical symptom (such as fatigue, difficulty breathing, loss of appetite, abdominal discomfort, nausea, and vomiting), and sleep problems or insomnia.
2. The Patient Health Questionnaire-9 (PHQ-9) Thai version, is a self-rating questionnaire to evaluate depression, consisting of nine questions. The score of each question employs a 4-point rating scale: 0 (never); 1(rarely); 2 (sometimes); 3 (always). The total score ranges from 0 to 27: 0–4 (no/minimal depression); 5–9 (mild depression); 10–14 (moderate depression); 15–19 (moderately severe); 20–27 (severe depression). The questionnaire indicates internal consistency; Cronbach’s alpha coefficient of 0.79; sensitivity of 0.53; and specificity of 0.98 [20]. The PHQ-9 seems to be an appropriate measurement to use for depression screening in cancer patients receiving radiotherapy, due to its excellent sensitivity (100%) and higher specificity (91.1%) [3].
3. Verbal Numerical Rating Scale (VNRS) was used to assess pain and the effect of pain administration. Patients rated their pain verbally on a numerical scale from 0 to 10; 0 (no pain); 1–3 (mild pain, slightly tolerable pain, lying down without pain, moving with slight pain); 4–6 (moderate pain, lying down with pain, moving with pain); 7–9 (severe pain);10 (intense pain, intolerable pain, worst pain imaginable). This questionnaire has the advantage of being simple and quick, demands no tool, and does not rely on intact motor skills [21].
4. The meaning in life questionnaire (MLQ) is a self-rating questionnaire, consisting of 10 questions. The score of each question employs a 5-point rating scale; 1 (absolutely untrue); 2 (quite untrue); 3 (unsure); 4 (quite true); 5 (absolutely true), with the total score ranging from 10 to 50. MLQ has 2 subscales; the search for meaning in life, and the presence of meaning in life. The search for meaning subscale determines how engaged and motivated respondents are in efforts to find meaning or deepen their understanding of meaning in their lives; whereas the presence of the meaning subscale determines how fully respondents feel their lives are of meaning [22, 23]. The higher the score determines that the respondent has more meaning in life. The questionnaire indicates internal consistency; Cronbach’s alpha coefficient of the search of meaning subscale and the presence of meaning subscale being 0.78 and 0.73, respectively [22].

## Statistical methods

All data were analyzed to describe the patients’ demographic, and depressive symptoms using the descriptive statistic method. The results were presented as proportions, frequency, median, and interquartile range (IQR). The analysis of the association between independent variables and depression symptoms used multiple logistic regression and was performed by R Foundation for Statistical Computing, version 4.1.2. All confidence intervals (CIs) were calculated at the 2-sided, 95% level.

## Results

### Demographic characteristics

Two patients with cancer refused to participate in this study, from this, 113 Thai outpatients with cancer participated and completed the questionnaires. The majority of them were female (61.1%), Buddhist (80.5%), and married (75.2%). The mean age was 56.2 ± 13.6 years (20–89 years old), and more than half of them (52.2%) had a low income (less than 5,000 baht per month). Most participants had an absence of a history of physical illness (excluding their cancer diagnosis) and psychiatric disorder (69.0%, and 96.5%, respectively). Among participants with physical illness, hypertension (62.9%), dyslipidemia (48.6%), and diabetes (28.6%) were the most common physical illnesses. The participants’ cancer staging was stage 2 and 3 (31.0%, and 32.7%, respectively). The location of cancer was breast (23.0%), cervix (11.5%), esophagus (11.5%), and other (54%) that included bladder, head and neck, thyroid, lung, lymphoma, penis, rectum, and brain. The median duration of cancer illness (IQR) was 5 (3–12) months, and fifty-nine participants (52.2%) had a history of being hospitalized for cancer treatment. Most participants rather felt satisfied with their health, perceiving their health as moderate to very good (83.2%). They identified being free of pain symptoms (47.8%), with only 13 (11.5%) and 2 participants (1.8%) reported experiencing moderate and severe pain, respectively. More than half of the participants (68.1%) had physical symptoms, and half of them had insomnia (50.4%) (Table 1).

**Table 1:** **Demographic characteristics of patients with cancer categorized by their status of depression (PHQ-9 score) (n = 113)**

Demographic characteristics	Total (n = 113)	Number (%) PHQ-9		Chi2 *P*-value
		No/Minimal (n = 87)	Mild (n = 26)	
**Gender**				0.528
Male	44 (38.9)	32 (36.8)	12 (46.2)	
Female	69 (61.1)	55 (63.2)	14 (53.8)	
**Status**				0.803^a^
Single	18 (15.9)	14 (16.1)	4 (15.4)	
Marital	85 (75.2)	66 (75.9)	19 (73.1)	
Divorce	10 (8.8)	7 (8.0)	3 (11.5)	
**Religion**				1
Buddhism	91 (80.5)	70 (80.5)	21 (80.8)	
Islamic	22 (19.5)	17 (19.5)	5 (19.2)	
**Education level**				0.469
Primary school	53 (46.9)	41 (47.1)	12 (46.2)	
Secondary school	30 (26.5)	21 (24.1)	9 (34.6)	
Bachelor’s degree or above	30 (26.5)	25 (28.7)	5 (19.2)	
**Location of cancer**				< 0.001^a^
Esophagus	13 (11.5)	4 (4.6)	9 (34.6)	
Cervix	13 (11.5)	10 (11.5)	3 (11.5)	
Breast	26 (23.0)	23 (26.4)	3 (11.5)	
Others (bladder, head and neck, thyroid, lung, lymphoma, penis, rectum, and brain)	61 (54.0)	50 (57.5)	11 (42.3)	
**Staging of cancer**				0.419
One	17 (15.0)	14 (16.1)	3 (11.5)	
Two	35 (31.0)	26 (29.9)	9 (34.6)	
Three	37 (32.7)	31 (35.6)	6 (23.1)	
Four	24 (21.2)	16 (18.4)	8 (30.8)	
**Duration of cancer illness** (months): Median (IQR)	5 (3, 12)	7 (3, 24)	3 (2.2, 5.5)	0.001^b^
**Having physical illness**				1
No	78 (69.0)	60 (69.0)	18 (69.2)	
Yes	35 (31.0)	27 (31.0)	8 (30.8)	
**Having psychiatric disorders**				1^a^
No	109 (96.5)	84 (96.6)	25 (96.2)	
Yes	4 (3.5)	3 (3.4)	1 (3.8)	
**Number of household member**				0.804
Less than 3	39 (34.5)	29 (33.3)	10 (38.5)	
3 or more	74 (65.5)	58 (66.7)	16 (61.5)	
**Having history of hospitalization**				0.023
No	54 (47.8)	36 (41.4)	18 (69.2)	
Yes	59 (52.2)	51 (58.6)	8 (30.8)	
**Perception of one’s health**				< 0.001
Very good / good	46 (40.7)	46 (52.9)	0 (0)	
Moderate	48 (42.5)	33 (37.9)	15 (57.7)	
Fair	19 (16.8)	8 (9.2)	11 (42.3)	
**Presence of physical symptoms**				< 0.001
No	36 (31.9)	36 (41.4)	0 (0)	
Yes	77 (68.1)	51 (58.6)	26 (100)	
**Presence of sleep problem or insomnia**				0.004
No	56 (49.6)	50 (57.5)	6 (23.1)	
Yes	57 (50.4)	37 (42.5)	20 (76.9)	
**Presence of pain**				< 0.001
No	54 (47.8)	50 (57.5)	4 (15.4)	
Mild	44 (38.9)	26 (29.9)	18 (69.2)	
Moderate to severe	15 (13.3)	11 (12.6)	4 (15.4)	

^a^ Fisher’s exact test; ^b^ Wilcoxon rank sum test

## Prevalence of depression

**Fig. 1:**
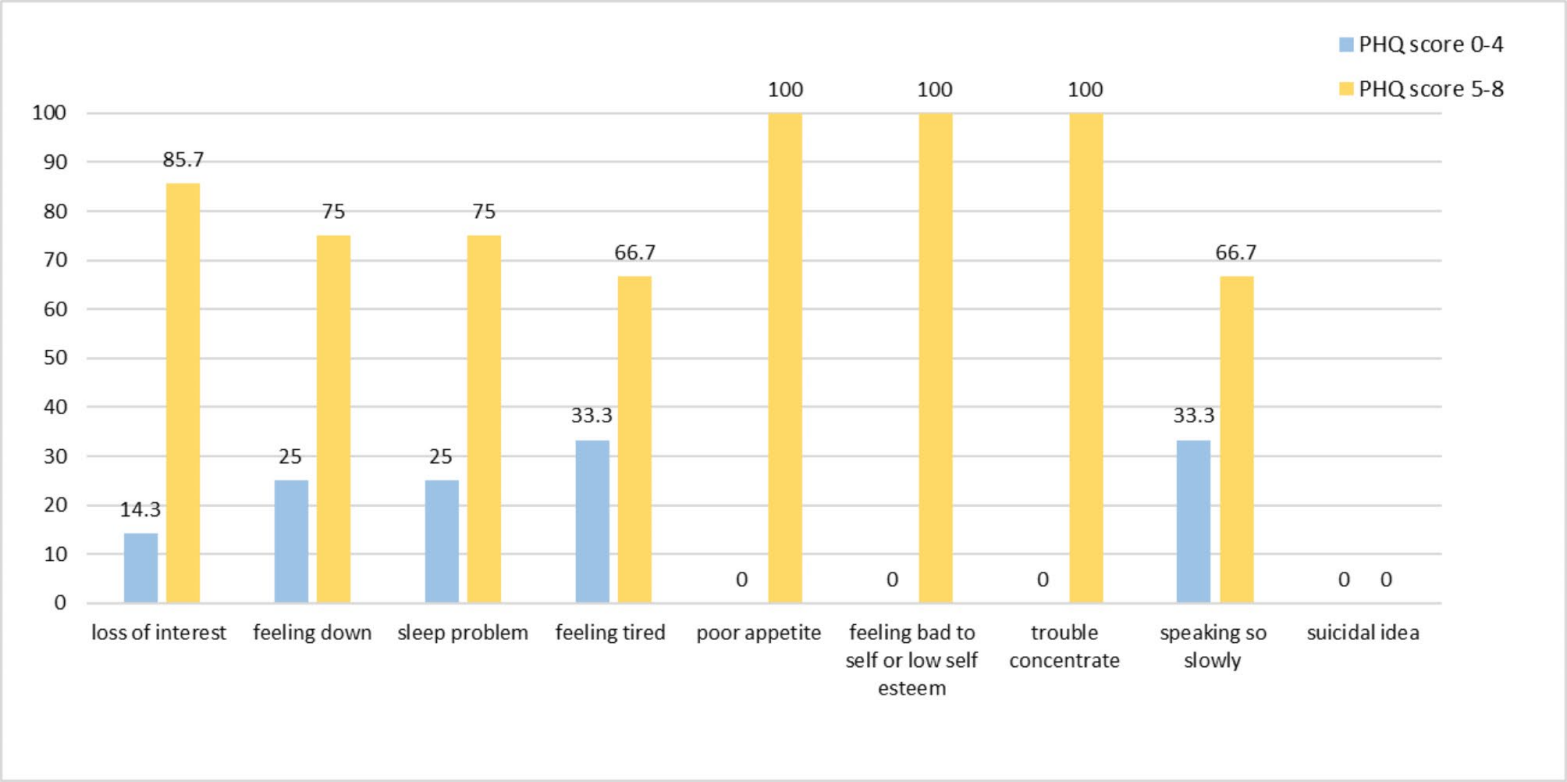
Frequency of depressive symptoms

The total PHQ-9 score of the 113 participants ranged from 0 to 8. Therefore, no participants had an experience of MDD (PHQ-9 score of nine or greater). Of all participants, eighty-seven (77.0%) reported a no/minimal depression (PHQ-9 score of four or less); whereas, the remaining (23.0%) had mild depression (PHQ-9 score from five to eight) (Fig. 1).

## Meaning in life

According to the meaning in life questionnaire, most of the participants had a high score in all subparts of meaning in life. All of them had the presence of meaning in life and were looking for something that made their lives feel meaningful or purposeful (Table 2). There were no differences between demographic characteristics and meaning in life scores (*p*-value > 0.05), except for self-perception of their general health, with those who felt more satisfied with their health having higher MLQ scores (*p*-value = 0.008) (data not shown in tables).

**Table 2:** Reported meaning in life of patients with cancer using MLQ (N = 113)

Subscale	Median	IQR
Presence of meaning in life		
I understand my life’s meaning.	5.0	4.0–5.0
My life has a clear sense of purpose.	5.0	4.0–5.0
I have a good sense of what makes my life meaningful.	5.0	4.0–5.0
I have discovered a satisfying life purpose.	5.0	4.0–5.0
My life has no clear purpose.	3.0	3.0–3.0
Search for meaning in life		
I am looking for something that makes my life feel meaningful.	4.0	4.0–5.0
I am always looking to find my life’s purpose.	4.0	4.0–5.0
I am always searching for something that makes my life feel significant.	4.0	4.0–5.0
I am seeking a purpose or mission for my life.	4.0	4.0–5.0
I am searching for meaning in my life.	4.0	4.0–5.0

## Association between demographic characteristics, pain, meaning in life and depression

Because there were no participants who had an experience of MDD in this study, we instead explored the factors that were associated with no/minimal and mild depression. The factors associated with having mild depression were the history of hospitalization, perception of one’s health, presence of physical symptoms, and pain. The participants who had no history of hospitalization had a higher rate of mild depression than those in the having history of hospitalization group: the adjusted odds ratio (AOR) was 3.3, and 95% CI of 1.02 to 10.7. The same was genuine when correlating them with those whose perception of one’s health was fair, and who had pain symptom; AOR (95%CI) was 6.9 (1.9, 25.7), and 6.0 (1.6, 22.9), respectively (Table 3).

**Table 3:** Final logistic regression of factors associated with mild depressive symptoms (N = 113)

Factors	Crude OR (95%CI)	Adjusted OR (95%CI)	*P*-value LR-test
**Having history of hospitalization**			0.037
Yes	Reference	Reference	
No	3.2 (1.3, 8.1)	3.3 (1.02, 10.7)	
**Presence of physical symptoms**			0.001
No	Reference	Reference	
Yes	N/A	N/A	
**Perception of one’s health**			0.002
Moderate to very good	Reference	Reference	
Fair	7.2 (2.5, 21.0)	6.9 (1.9, 25.7)	
**Presence of pain**			0.004
No	Reference	Reference	
Yes	7.4 (2.4, 23.4)	6.0 (1.6, 22.9)	

**Note:** OR, odds ratio; N/A, not applicable.

Most of the participants had a high score in all subparts of meaning in life. Based on this dataset, the relationship between meaning in life and depression could not be analyzed.

## Discussion

This is the first study from Southern Thailand that purposed to explore the prevalence of depression including associated factors for patients with cancer receiving radiotherapy. The prevalence of depression discovered was no MDD, with all participants having no/minimal or mild depression. Furthermore, the associated factors related to mild depression were a history of hospitalization, a perception of one’s health, the presence of physical symptoms, and pain. These findings are different from those of prior reports using PHQ-9 from India [24], Malaysia [25], Canada [26], and meta-analytical pooled prevalence of depression defined by the International Classification of Diseases (ICD) or DSM criteria that found the prevalence of MDD ranged from 14.3 to 70.0% [27, 28]. It also differed from a recent study among Thai patients with cancer who received radiotherapy from the central regions of Thailand; in that, the reported prevalence of MDD was 12.1% [3]. A potential explanation for these discrepancies may be due to different study instruments and characteristics of the population; such, as ethnicity, age group, socioeconomic status, family and social support; type or location of cancer, staging, and duration of cancer illness [29–31].

Most participants in this study had a high score in all subparts of meaning in life and were satisfied or appreciated with their health. Indeed, in this study, patients with cancer who had higher MLQ scores were more likely to satisfy with their general health. As mentioned above, this could be because the “narrative” model has been used with patients with cancer in this hospital to change the meaning of cancer into something more positive and to search for meaning in their life. Meaning in life may be viewed as coherent to the patient’s sense of relevance, allowing them not only to anchor or standpoint themselves in reality, but also to analyze new purposes in which to invest for the remaining life [19, 32]. Moreover, most participants (101/113, 89.4%) stayed at the Yensira building over their course of radiotherapy and on the day of data collection. The building provided basic care; including, life safety, and feelings of comfort by having friends with cancer as well as causing an exchange of attitudes in life, life solutions, and not feeling lonely. All of these may be the main psychosocial and spiritual factors that promote happiness, and life satisfaction, prevent feelings of sadness as well as make the patients with cancer perceive themselves as a person of identity, values, and connected to people in society. Because a prior study identified that life satisfaction was one of the most vigorous factors related to depression, and mediated the capability of prognostic awareness of depression. Moreover, low life satisfaction increased the risk of depression with an odd ratio of 3.01 (95% CI = 2.37,3.82) [26].

Concerning the associated factors with mild depression, our findings were similar to the prior studies reporting no effect of gender, age, marital status, occupation, education, or cancer location including the staging of illness on the presence of depressive symptoms [3, 28]. However, this study also found the factors associated with mild depression were the presence of physical symptoms and pain. Pain can produce psychological and psychiatric distress, which may be a major source of suffering for patients with cancer and their relatives [27]. It is also associated with disability, impaired physical activity, and poor social interaction [8, 33]. Moreover, the prior study identified that most Thai patients with palliative cancer, along with the general Thai population in Southern Thailand, preferred End-of-life care to be free of distressing or uncomfortable symptoms, such as pain [18, 34]. Therefore, to encourage good quality of life and happiness among patients with cancer, healthcare professionals should be aware of and manage the suffering symptoms, including pain, adequately.

In this study, cancer patients with a history of hospitalization had lower rates of mild depression. In our opinion, having a history of hospitalization may allow patients with cancer to experience adaptation or cope with life-threatening stress more so than others. After surviving a life crisis, they may perceive the remaining of their life as synonymous with life gains or rewards. Additionally, during hospitalization, necessary treatments or psychological interventions may be arranged and met with optimism. Therefore, the outlook for the rest of life is more positive and helps the patient with cancer achieve a sense of acceptance of their illness and possible death [19, 26].

Finally, evidence-based guidelines on the management of depression in cancer care recommend optimal support and care to prevent depression; early detection and severity assessment of depression; and adequate and effective management using antidepressant medication and psychological interventions [17, 35, 36]. The associated factors identified in this study may be used to design an intervention and guide clinical practice for the prevention, early diagnosis, and management of depression in patients with cancer. For palliative care providers, in this study, only 21.2% of participants were patients with palliative cancer, although this is a small number compared to the rest of the participants. However, in our opinion, the result of this study may be useful and encouraging for palliative care providers to continue their efforts to detect, reduce, and relieve both pain and depressive symptoms of their cancer patients. Additionally, it also reaffirms that trying to enable meaningful lives of our cancer patients early is crucial to making terminal palliative care more convenient and easier.

## Strengths and limitations

To our knowledge, this was the only study on this topic having been operated in Southern Thailand during the past decade. Even though there were no cancer patients with MDD obtained from this study, we tried to search for the relationship between factors and minor depression. These findings may be novel knowledge for the prevention of early or low-severity depression. Despite regular briefings with clinical nurses who acted as gatekeepers and helped recruit participants, they might have selected those with more stable physical and mental conditions and seemed able to participate in the study. This might result in selection bias or the limited number of participants and then lower rates of depression found among the participants. Additionally, this may also be an effect of the potentially weak measurement of depression. Nevertheless, the questionnaire has been tested elsewhere with good reliability and used widely. This study was also quantitative, and its sample size was limited to only cancer outpatients receiving radiotherapy in lower Southern Thailand. Most participants were female in gender, had a low income, and were in old age groups. Hence, these results may not demonstrate or generalize the predicament or condition of cancer patients in all age groups, all economic statuses, or the whole country fairly. Henceforth, future studies should include a larger number of cancer outpatients with gender, age group, and economic status differences from other hospitals in Thailand; in other words, a multi-center study that purposes to identify this research topic should be employed. Moreover, such research should operate an in-depth methodology that is adept at analyzing specific disorders or a more qualitative approach.

## Conclusion

In this study, all cancer participants who received radiotherapy had either no/minimal or mild depression. No participants had major depression. Most participants had meaning in their life; however, over half of them still experienced pain and insomnia. To optimize the quality of life, and prevent depression, physical symptoms, and pain should ensure they receive adequate management. Additionally, feeling meaningful in life, and satisfaction in one’s health should also be of concern.

## Data Availability

The datasets used and/or analyzed during this current study can be made available by the corresponding author upon reasonable request.

## Abbreviations

MLQ: The meaning in life questionnaire
PHQ-9: The Patient Health Questionnaire-9
VNRS: The questionnaire of Verbal Numerical Rating Scale
